# Supplementary material for: Uninterrupted HIV treatment for women: Policies and practices for care transitions during pregnancy and breastfeeding in Côte d’Ivoire, Lesotho and Malawi
Source: PLoS One. 2021 Dec 2;16(12):e0260530. doi: 10.1371/journal.pone.0260530 (PMC8638956; doi:10.1371/journal.pone.0260530)
Supplement: S1 Appendix — (PDF) [file pone.0260530.s001.pdf]

Appendix C: Semi-structured interview guide for key informant interviews

Participant Identification code	
Date (dd/mm/yyyy)	
Country	
Interviewer initials	

INTRODUCTION

As noted in the consent form, we are interested in understanding more about HIV health systems in your country specifically related to how and when women living with HIV transition from antenatal care (ANC) and prevention of mother-to-child transmission (PMTCT) services back to adult services following a pregnancy. We are also interested in how women living with HIV who are taking antiretroviral therapy (ART) transition to ANC and PMTCT services when they become pregnant. As you are aware, high rates of loss to follow-up have been noted at the time when women are transitioned from MCH to ART services following a pregnancy. We are interested in your knowledge about this issue and appreciate any and all feedback and input you are willing to provide.

First, we will start off by asking you some basic demographic information. Please note that we will not ask for your name or any information that could identify you.

Question 1: What is your sex?

a. Male

b. Female

c. Prefer not to answer

Question 2: What is your age?

a. 20-30

b. 31-40

c. 41-50

d. 51-60

e. 60+f. Prefer not to answer

Question 3: What sector do you work in?

a. Governmental agency (ministry, province, district, other) (Specify type)

b. Academia/research organization

c. Non-governmental organization (NGO)

d. Implementing partner

e. Other (describe)

f. Prefer not to answer

Question 4: How many years have you worked in your current position?

a. 0-2

b. 3-5

c. >5

d. Prefer not to answer

Question 5: Please describe the capacity in which you provided input on the development of national guidelines on PMTCT and/or ANC for women living with HIV? (Please describe your activities/responsibilities but you do not need to tell your exact title or role if you do not wish to – we will not publish or share the information about your title outside of the study).

- Did you help develop the guidelines? Review them? Edit them?
- Did you participate in meetings through this process? What kind of meetings?
- How long have you been part of developing the national guidelines?

Question 5.1: Describe the capacity in which you oversee or support the **implementation** of national guidelines on PMTCT and/or ANC for women living with HIV? (As previously, please describe your activities/responsibilities but you do not need to tell your exact title or role if you do not wish to – we will not publish or share the information about your title outside of the study).

- Do you support the implementation at district level? At site level?
- Were you involved with any of the trainings with healthcare staff? If so, at what capacity (developing training materials, training directly, etc.)?
- How have you continued to provide support on implementation of the guidelines after the initial role out?

The following questions relate to the current national guidelines – those that are on paper in official guidance documents. Later on, I will ask you about what is current practice or standard of care that may not be in official guidelines.

Question 6: Please describe how the current national guidelines for care of pregnant women living with HIV were developed including what stakeholders were involved and what information was taken into consideration when creating the guidelines.

- Based on global guideline changes (e.g., WHO)? National level experiences?
- Based on any evaluations or data collected?

Question 7: Please describe how women living with HIV who are on ART are identified as having a pregnancy per the current national guidelines (i.e. pregnancy testing procedures for women receiving HIV care/ART).

Question 8: Describe the current national guidelines for the care of women on ART who become pregnant.

- For instance, please describe how and when they should be transitioned to ANC/maternal child health (MCH) services for pregnancy care.

Question 8.1: According to the guidelines, are women on ART at the time that they become pregnant able to receive ART in ANC/MCH services or do they continue to receive HIV care and ART from adult care clinics?

Question 9: Describe the current national guidelines related to what happens to pregnant women who are identified as having HIV during a pregnancy with regard to where they receive HIV care and ART during pregnancy. (i.e., ANC/maternal child health (MCH) or adult HIV clinics)

Question 10: If applicable, according to the guidelines, for how long should women living with HIV receive their HIV/ART care within ANC/maternal child health (MCH) services after delivery before returning back to general adult HIV care for their HIV care only?

- How long do HIV uninfected mothers continue care at ANC/MCH?
- How long do HIV-infected mothers continue care at ANC/MCH? Does this include HIV care or is that solely in the adult ART clinic?

Question 10.1: According to the guidelines, if women continue to receive HIV care and ART within the ANC/MCH after delivery, are the mother's HIV services integrated with care for the infant (i.e. do they occur in the same clinic)?

Question 10.2: Are HIV/ART care for mothers and infant services offered on the same day at the clinic or do women make separate visits for their own care/ART pick-up and infant follow-up visits?

Question 11: To follow up on the previous question, on a scale of 1 to 10, where 1 is not implemented at all, and 10 is completely implemented, to what extent have these guidelines been implemented as planned across the country? (Please feel free to describe any regional variations or other additional contextual information).

- Describe the role out plan (who was involved, how long did it take, at what level were trainings done)
- What went well with implementation?

Question 11.1: What is your assessment based on? For instance, please describe data, evaluations and/or observations from health facilities that have informed your assessment.

Question 12: What challenges were experienced with initially implementing these guidelines?

- Government issues? Stakeholder "push back"?

Question 12.1: What challenges continue to exist with implementing the guidelines?

Question 13: If there are not current guidelines on transitioning women (meaning formal guidance in documents providing instructions how and/or when to transition), are there plans to include specific guidance around transitioning women living with HIV into and out of PMTCT/ANC/MCH back to adult HIV care in future guidelines? Please provide any information about this and what the timeline may be.

Question 14: Please describe the current guidelines regarding mother-infant pair tracking of HIV-exposed infants following delivery.

Question 14.1: Are there any plans to update or change current guidelines for mother-infant, including updating monitoring tools? If yes, please describe.

The following questions relate to any differentiated service delivery (DSD) models, demonstration or pilot project and evaluations or studies, that are currently underway, are planned or have been completed in [country] examining or aimed at improving transition of women living with HIV to/from PMTCT and adult services. This relates to current practice or standard of care that may not be in official guidelines.

Question 15: Please describe [country] current PMTCT and/or ANC for women living with HIV practices if it differs from the official guidelines you described previously.

- What about for transitioning women living with HIV to general adult ART clinics/services and back to PMTCT at the start of a new pregnancy?
- What is currently being done at a clinic level that is different from what is in the official guidelines?

***Question 16*:** Are there any differentiated service delivery models in-country that you are aware of that target care of pregnant women living with HIV? If yes, please describe.

- By DSD, I mean different models of care for pregnant women living with HIV which may include multi-month scripting, fewer ART clinic visits, etc.

Question 16.1: Are any DSD models part of national guidelines? If yes, indicate which ones.

Question 16.2: Do any DSD models specially target the transition of women on ART into and out of PMTCT/ANC/MCH services?

Question 16.3: Are there any plans to add DSD models for pregnant women on ART to the national guidelines?

Question 17: What models of care or interventions, if any, should be implemented to improve transition for women on ART into and out of PMTCT/ANC/MCH services?

Question 18: What strategies or interventions do you think would work best in transitioning of women living with HIV to/from PMTCT and adult services? This could either be what is currently in the guidelines or approaches/strategies that you think should be included as "best practices".

Question 19: How do you think the current guidelines could be improved related to transition?

Question 20: Those are all the questions I have for you today. Are there any other further comments you would like to provide?

Thank you for your time!
